# Cigarette smoke activates the parthanatos pathway of cell death in human bronchial epithelial cells

**DOI:** 10.1038/s41420-019-0205-3

**Published:** 2019-08-05

**Authors:** Lisa Künzi, Gregory E. Holt

**Affiliations:** 1 Division of Pulmonary, Allergy, Critical Care and Sleep Medicine, Department of Medicine, University of Miami, Miami, FL USA; 2 Division of Pulmonology, Department of Medicine, Miami VA Medical Center, Miami, FL USA

**Keywords:** Chronic obstructive pulmonary disease, Necroptosis

## Abstract

Tobacco smoke negatively affects human bronchial epithelial (HBE) cells and is directly implicated in the etiology of smoking related respiratory diseases. Smoke exposure causes double-stranded DNA breaks and DNA damage activates PARP-1, the key mediator of the parthanatos pathway of cell death. We hypothesize that smoke exposure activates the parthanatos pathway in HBE cells and represents a cell death mechanism that contributes to smoking related lung diseases. We exposed fully differentiated, primary HBE cells grown at the air liquid interface to cigarette smoke and evaluated them for parthanatos pathway activation. Smoke exposure induced mitochondrial to nuclear translocation of Apoptosis-Inducing Factor (AIF) and Endonuclease G (EndoG) within the first three hours characteristic of the parthanatos pathway. Exposing cells to an increasing number of cigarettes revealed that significant activation of the parthanatos pathway occurs after exposure to higher levels of smoke. Use of the specific PARP-1 inhibitor, BMN673, abrogated the effect of smoke induced activation of the parthanatos pathway. Smoke-mediated activation of the parthanatos pathway is increased in HBE cells originating from habitual smokers compared to non-smokers. This suggests that chronic smoke exposure leads to an increase in smoke-mediated activation of the parthanatos pathway and implicates its contribution in the pathogenesis of smoke-related lung diseases.

## Introduction

Cigarette smoke exposure is the major risk factor for chronic obstructive pulmonary disease (COPD), a disease characterized by airway remodeling^1^. Evaluation of lungs from patients with COPD finds the predominant histologic and molecular changes to occur within bronchial epithelial cells^2^. Smoke induced cell death of airway cells has been hypothesized^3^ to play a role in both airway remodeling and inflammation contributing to disease progression^4^. Although smoke has been shown to cause apoptosis of HBE cells^5–7^, apoptosis is classically considered a non-inflammatory form of programmed cell death^8^. Necrotic cell death is pro-inflammatory^9^ and exposure of bronchial epithelial cell lines to cigarette smoke extract (CSE) induced histologic cell death suggestive of necrosis^10^. This implies a mechanistic link between smoke exposure mediated HBE cell necrosis and the chronic inflammation associated with airway remodeling. This theoretical association however is unproven and the mechanisms responsible for cigarette smoke induced airway remodeling remain largely unknown^1^.

Cigarette smoke contains high levels of reactive oxygen species (ROS)^11,12^ and produces additional ROS upon exposure to human bronchial epithelial (HBE) cells^10,13^. Oxidative stress damages DNA by producing peroxynitrite (ONOO^−^) from the reaction of superoxide anions and nitric oxide^14^ and correlates with increased airway epithelial cell apoptosis, pro-inflammatory signaling and DNA oxidation^15^. Smoke mediated oxidative damage of the genome produces DNA strand breaks^16^ in HBE cells^17^. DNA strand breaks activate the Poly(ADP-ribose) polymerase-1 (PARP-1) protein^18^ that analyzes the extent of DNA damage to decide between DNA repair or programmed cell death^19^. Manageable levels of DNA damage initiate DNA repair by PARP-1 mediated ADP-ribosylation of itself and associated proteins required for base-excision repair^20^. However, excessive genetic damage due to overwhelming DNA damage overactivates PARP–1 leading to activation of a distinct programmed cell death pathway called parthanatos^21^.

**Fig. 1:**
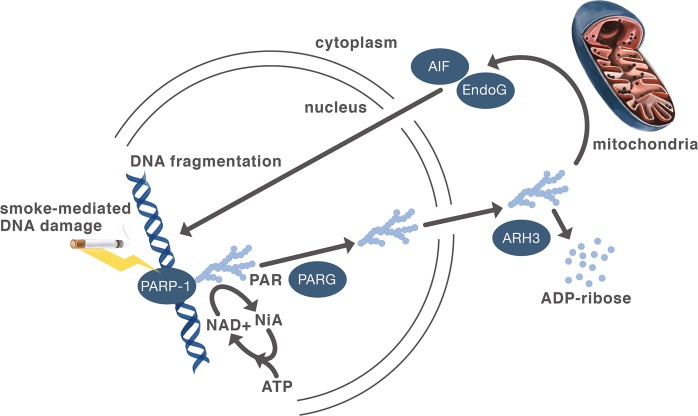
Schematic of proposed cigarette smoke activation of the parthanatos pathway of cell death. PARP–1 recognition of smoke-mediated DNA strand breaks activates itself to poly-adenosylate (PAR) itself and other nuclear proteins^20^. Extensive DNA damage resulting in PARP–1 overactivation produces branched chains of PAR^21^. Poly (ADP-ribose) glycohydrolase (PARG) releases PAR by cleavage that unless hydrolyzed by ADP-ribosylhydrolase 3 (ARH3)^37^ induces mitochondrial proteins Apoptosis-Inducing Factor (AIF) and EndonucleaseG (EndoG) to translocate to the nucleus causing genomic DNA fragmentation^21^

The parthanatos pathway (Fig. 1) was initially described in excitotoxic neuronal cell death associated with strokes and neurodegenerative diseases^22^. These diseases cause oxidative stress in neurons that also produce peroxynitrite (ONOO^−^) from the reaction of superoxide anions and nitric oxide causing DNA damage with subsequent PARP-1 activation. Overactivation of PARP-1 produces long chain, branched polymers of poly (ADP-ribose) that induce nuclear translocation of the mitochondrial proteins Apoptosis-Inducing Factor (AIF) and Endonuclease G (EndoG)^21,23^. In the nucleus, AIF orchestrates cleavage of genomic DNA into large fragments resulting in cell death. Of the enlarging number of programmed cell death pathways^9^, parthanatos is uniquely characterized by its dependence on PARP-1 overactivation mediated AIF translocation to the nucleus^21^. Since its initial discovery in neuronal cell death, the parthanatos pathway has been implicated in other diseases including diabetes mellitus^24^, pulmonary fibrosis and acute respiratory distress syndrome^21,25,26^.

Because HBE cell production of ROS after smoke exposure is similar to neuronal production of ROS after a stroke that activates the parthanatos pathway, the aim of this study was to evaluate whether cigarette smoke activates the parthanatos pathway in HBE cells. Using primary, fully matured HBE cells grown at the air liquid interface exposed to mainstream smoke from tobacco cigarettes, this study evaluated activation of the parthanatos pathway by measuring the mitochondrial to nuclear translocation of AIF and EndoG. Since the parthanatos pathway is considered a form of regulated necrosis associated with inflammation^9^, finding smoke activates parthanatos in HBE cells could provide a mechanistic link between smoke exposure and the chronic inflammation that characterizes airway remodeling in COPD.

## Results

### Cigarette smoke exposure induces translocation of AIF and EndoG to the nucleus

**Fig. 2:**
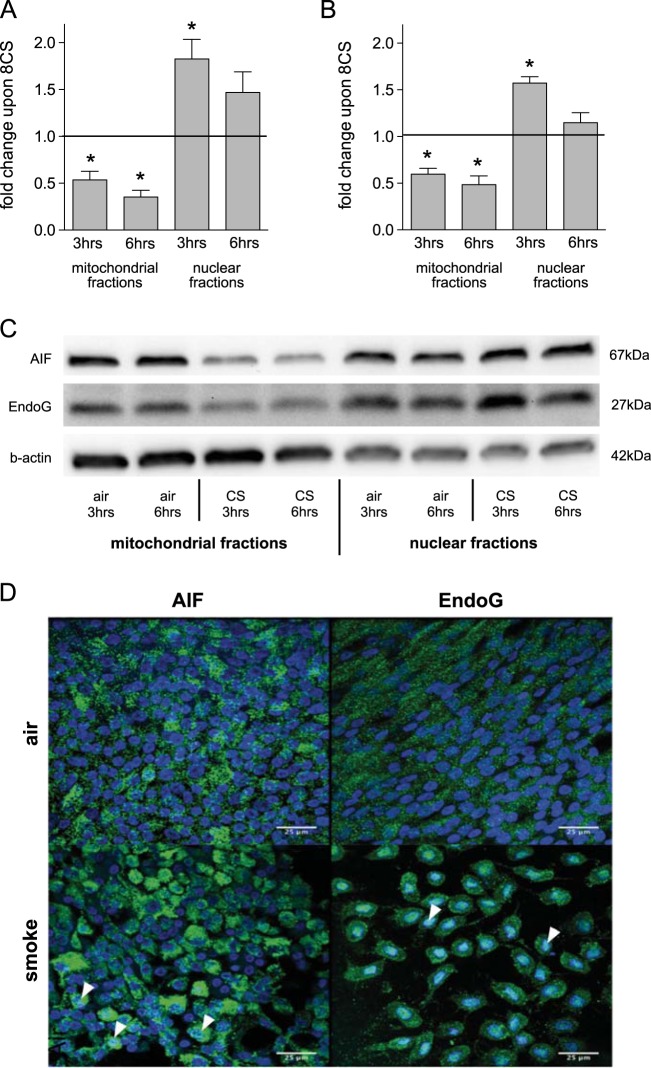
Cigarette smoke exposure activates the parthantos pathway in HBE cells. Nuclear translocation of mitochondrial proteins AIF (**a**) and EndoG (**b**) in HBE cells after cigarette smoke exposure. HBE cultures from non-smoker (*n* = 5) were exposed to smoke of eight cigarettes or an equivalent volume of clean air and fractionated 3 and 6 h after exposure, respectively. Protein levels in mitochondrial and nuclear fractions were determined by western blot (**c**), normalized to β-actin for protein loading and expressed as fold change in smoke exposed cells compared to their corresponding clean air control; mean + SEM, *p ≤ 0.05. **d** Fluorescence immunohistochemistry of HBE cells upon cigarette smoke exposure showing nuclear translocation of AIF and EndoG. HBE cells were exposed to smoke of eight cigarettes or an equivalent volume of clean air. Cells were stained for AIF (green, left panels) and EndoG (green, right panels) and counterstained for nuclei with DAPI (blue). Nuclear localization of AIF and EndoG resulted in a light blue staining (arrow heads)

**Fig. 3:**
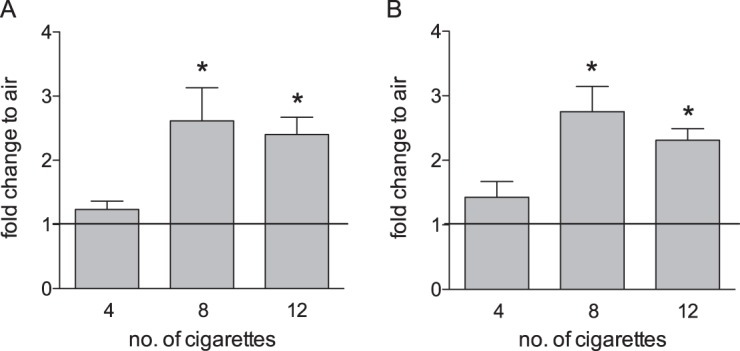
Cigarette smoke induced activation of parthanatos occurs at higher smoke exposures. Nuclear translocation of AIF (**a**) and EndoG (**b**) in HBE cultures three hours after exposure to 4, 8 or 12 cigarettes was determined by western blot analysis (*n* = 4–6). Data are normalized to β-actin for protein loading and presented as fold change, where the normalized quantity of each protein for each subcellular fraction in smoke exposed cultures is divided by the quantity of the identical protein in the same subcellular fraction in the air exposed cultures originating from the same donor; mean + SEM, *p ≤ 0.05

Translocation of AIF to the nucleus is the defining characteristic of the parthanatos pathway^21^ and is accompanied by EndoG translocation^23^. Acute exposure of primary, fully matured HBE cells to smoke from eight cigarettes significantly decreased AIF and EndoG in mitochondria and increased both proteins in the nucleus at three and six hours after exposure (Fig. 2; mitochondrial fractions: *p*_AIF@3 h_ = 0.007, *p*_AIF@6 h_ < 0.001, *p*_EndoG@3 h_ = 0.003, *p*_EndoG@6 h_ = 0.005; nuclear fractions: *p*_AIF@3 h_ = 0.017, *p*_AIF@6 h_ = 0.099, *p*_EndoG@3 h_ = 0.001, *p*_EndoG@6 h_ = 0.240). Fluorescence Immunohistochemistry of HBE cells confirmed accumulation of both AIF and EndoG in the nucleus of smoke exposed cells (Fig. 2d) consistent with activation of the parthanatos pathway. HBE cells were exposed to an increasing dose of smoke ranging from four to twelve cigarettes and parthanatos activation was assessed three hours after exposure by comparing nuclear AIF and EndoG levels to corresponding clean air controls. This revealed significant parthanatos activation occurs with exposure to eight or greater cigarettes, but not after four cigarettes (Fig. 3; AIF: *p*_4CS_ = 0.170; *p*_8CS_ = 0.026; *p*_12CS_ = 0.006; EndoG: *p*_4CS_ = 0.172; *p*_8CS_ = 0.0065; *p*_12CS_ = 0.002).

**Fig. 4:**
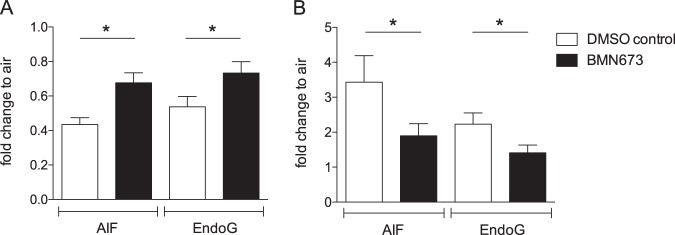
Use of PARP-1 inhibitor BMN673 decreases smoke-mediated activation of the parthanatos pathway. Mitochondrial to nuclear translocation of AIF and EndoG was quantified by western blots of HBE cultures three hours after exposure to smoke of eight cigarettes. AIF and EndoG in mitochondrial (**a**) and nuclear (**b**) fractions was quantified by western blotting and normalized to β-actin for protein loading. Data are presented as fold change, where the normalized quantity of each protein for each subcellular fraction in smoke exposed cultures is divided by the quantity of the identical protein in the same subcellular fraction in the air exposed cultures originating from the same donor. AIF and EndoG translocation in BMN673 treated cultures were compared to their corresponding DMSO control; mean + SEM, *n* = 6, *p ≤ 0.05

Since PARP-1 activation is a prerequisite to initiate the parthanatos pathway^23^, we treated HBE cultures with the selective PARP-1 inhibitor BMN673 currently in clinical trials for cancer treatment^27^ and exposed the cells to mainstream smoke. Use of BMN673 in HBE cultures significantly reduced smoke-mediated nuclear translocation of AIF and EndoG compared to the DMSO treated controls consistent with inhibition of parthanatos activation (Fig. 4; BMN673 decreases AIF and EndoG loss from mitochondria: *p*_AIF_ = 0.010, *p*_EndoG_ = 0.009; and reduces nuclear increase: *p*_AIF_ = 0.047, *p*_EndoG_ = 0.028).

### HBE cells from habitual smokers exhibit increased activation of the parthanatos pathway

**Fig. 5:**
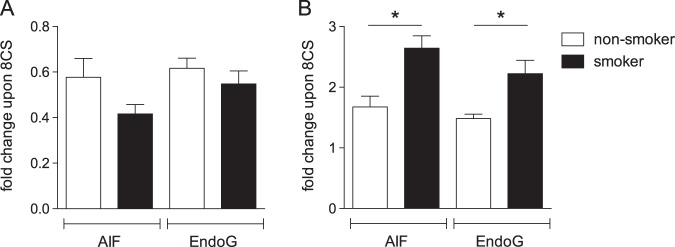
Nuclear translocation of AIF and EndoG in HBE cells from non-smokers (*n* = 7) and smokers (*n* = 14). Cultures were exposed to the smoke of 8 cigarettes or an equivalent volume of clean air, followed by isolation of mitochondrial and nuclear fractions three hours post exposure treatment. AIF and EndoG in mitochondrial (**a**) and nuclear (**b**) fractions were quantified by western blotting and normalized to β-actin. Data are presented as fold change, where the normalized quantity of each protein for each subcellular fraction in smoke exposed cultures is divided by the quantity of the identical protein in the same subcellular fraction in the air exposed cultures originating from the same donor; mean + SEM, *p ≤ 0.05

**Fig. 6:**
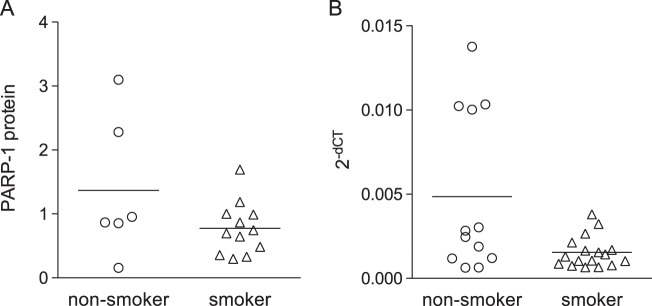
RT-PCR and western blot analysis of PARP-1 expression in HBE cells from primary, fully matured HBE cells. **a** RT-PCR of PARP-1 normalized to GAPDH in HBE cells from non-smokers (*n* = 12) and smokers (*n* = 17). **b** Western blot analysis of PARP-1 protein expression normalized to β-actin in HBE cells from non-smokers (*n* = 6) and smokers (*n* = 12). Plotted individual data points with line representing mean

Chronic smoke exposure is known to induce epigenetic changes to bronchial epithelial cells altering their subsequent response to smoke exposure^28^. We evaluated HBE cells classified as non-smoker or smoker based on clinical history to determine if chronic smoke exposure during life altered smoke-mediated activation of the parthanatos pathway. HBE cells from both groups were exposed to the smoke of eight cigarettes and evaluated for AIF and EndoG translocation by western blotting. Interestingly, HBE cells from smokers exhibited higher nuclear translocation of AIF (*p* = 0.006) and EndoG (*p* = 0.032) than cells from non-smokers (Fig. 5) consistent with an increase in activation of the parthanatos pathway in cells derived from habitual smokers. Comparison of mitochondrial levels of AIF revealed a trend towards less AIF (*p*_AIF_ = 0.065) in smoker’s versus non-smoker’s HBE cells, but similar EndoG levels (*p*_EndoG_ = 0.444). Comparison of PARP-1 expression in HBE cells from non-smokers and smokers based on mRNA and protein levels revealed no clear differences between the two groups (Fig. 6; *p*_mRNA_ = 0.0882, *p*_protein_ = 0.607).

## Discussion

To our knowledge smoke-mediated activation of the parthanatos pathway in primary HBE cells has not been previously reported. First described in neuronal cell death, oxidative stress induced by both neuronal excitotoxicity^29^ and smoke exposure^13,16,17^ leads to PARP-1 activation and initiation of the parthanatos pathway. The finding of increased nuclear translocation of AIF and EndoG in HBE cells from smokers compared to non-smokers suggests it may play a role in smoke-related lung diseases.

The parthanatos pathway is uniquely characterized by PARP-1 mediated translocation of AIF to the nucleus^21^. Our data from primary, fully differentiated HBE cells show AIF and EndoG translocate to the nucleus within a three hour time frame after smoke exposure. These data compare favorably to published reports where 80% of AIF translocated within three hours after exposure to DNA damage inducing agents^30^. Use of the PARP-1 inhibitor, BMN673, reduced AIF and EndoG translocation after smoke exposure, confirming the role of PARP-1 activation in smoke induced activation of the parthanatos pathway in primary HBE cells.

Parthanatos is a programmed cell death pathway classified as a form of regulated necrosis leading to swelling and rupture of cells and organelles resulting in inflammation^9^. The release of cellular contents results in signals called Danger-Associated Molecular Patterns (DAMPs) that induce the production of pro-inflammatory cytokines, e.g. IL-6, IL-8, from HBE cells that recruit pro-inflammatory immune cells^31^. Smoke is known to induce airway inflammation via IL-6 and IL-8 production and recruit inflammatory cells including neutrophils, however the mechanism causing the release of these cytokines has not been well described^32^. We hypothesize that smoke-mediated activation of the parthanatos pathway of programmed cell death produces inflammation that if chronic, results in airway remodeling. Evidence for this is suggested by the finding that in COPD patients increasing PARP-1 activity correlates with disease severity^33^. Thus, smoke-mediated activation of the parthanatos pathway may represent one mechanism directly linking smoke exposure to the chronic inflammation of bronchial epithelia seen in smoking related diseases.

Smoke exposure leads to significantly more AIF and EndoG nuclear translocation in HBE cells from smokers than non-smokers. This finding suggests a link between the parthanatos pathway and smoke-related diseases of the bronchial epithelia. Evaluation of PARP-1 levels between the two populations in this study did not provide a simple answer. However, the consequence of the increased activation of the parthanatos pathway in HBE cells from habitual smokers may lie in balancing genome integrity with airway inflammation. Chronic toxin exposure often alters a cell’s response to repetitive exposures by attenuating the negative response^34^. However, in this case, chronic smoke exposure increased smoke-mediated activation of the parthanatos pathway of cell death in HBE cells. This counterintuitive finding may be explained by the fact that one role of the parthanatos pathway is to remove cells with severe genotoxic damage to prevent formation of dysfunctional or tumorigenic cells. Thus, increased activation of the parthanatos pathway in habitual smokers’ HBE cells exposed to smoke may represent an epigenetic adaptation to chronic smoke exposure aiming to protect the integrity of the genome over individual cell survival.

The parthanatos pathway is classically initiated after PARP-1 overactivation occurs from overwhelming DNA damage. Theoretically, this protects the organism from cells whose DNA has been so significantly damaged that repair of the genome would lead to unacceptably high mutation rates. In this study, the parthanatos pathway was activated only after exposure of primary HBE cells to higher levels of cigarette smoke. Consistent with this finding, previous studies reported the degree of DNA damage correlated with the exposure of HBE cells to increasing concentrations of CSE^17^. This supports the role of the parthanatos pathway in protecting the organism against genomic mutations from exposure to increased toxin levels.

Currently, there is little known about the role of the parthanatos pathway in smoke-related lung disease. Inhibiting parthanatos activation could block the associated inflammation, but increase genomic mutations^10^. Alternatively, increasing parthanatos activation to remove cells with significant smoke induced DNA damage could prevent incorporation of genetic mutations, but lead to increased inflammation. Understanding the role the parthanatos pathway plays in HBE cell response to smoke exposure is therefore important before contemplating interventions to influence its activation as a strategy to treat smoke-related lung disease.

## Materials and methods

### Cell culture

**Fig. 7:**
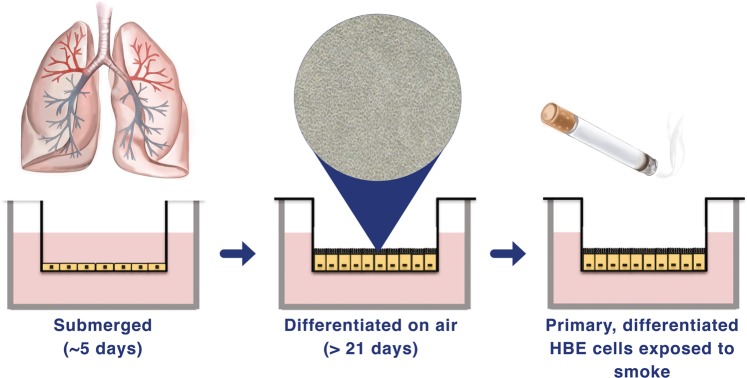
Fully differentiated primary human bronchial epithelial (HBE) cells grown at the air liquid interface (ALI). HBE cells from lungs rejected for organ donation are grown in a two-chamber culture system where they differentiate for at least 21 days at the ALI into mature bronchial epithelia. HBE cells are exposed to the mainstream smoke from tobacco cigarettes by a smoke robot closely mimicking in vivo conditions

Differentiated, primary HBE cells grown at the air liquid interface (ALI; Fig. 7) were prepared as previously described^35,36^. Briefly, human tracheobronchial epithelial cells were harvested from lungs rejected for transplantation provided by the University of Miami Life Alliance Organ Recovery Agency for research with appropriate consent and in accordance with the declaration of Helsinki. HBE cells were grown to confluence on collagen-coated dishes in Bronchial Epithelial Growth Medium. Passage 1 or 2 cultures were seeded onto collagen IV (C7521, Sigma, St Louis, MO) coated 12 mm Transwell inserts (3460, Corning Costar Corporation, Cambridge, MA) and grown in ALI media to 80% confluence before exposing the apical surface to air. Apical surfaces were washed every other day with Dulbecco’s phosphate-buffered saline (DPBS; 45000-430, VWR, Radnor, PA). After four weeks, cultures were fully differentiated with ciliated, goblet and basal cells as evidenced by beating cilia and mucus production. Smoking status was recorded at the time of organ donation from family and medical records.

### Cigarette smoke exposure

HBE cells were exposed to mainstream smoke from 3R4F University of Kentucky reference cigarettes (Lexington, KY) using a VC-10 Smoking Robot (Vitrocell, Waldkirch, Germany) in accordance with ISO 3308 standards (puff duration 2 s, hold time 0 s, exhaust time 8 s, puff volume 35 mL, frequency 60 s, 6 puffs per cigarette). Control cells received an equivalent volume of clean air.

### Immunohistochemistry

Cells were fixed in 4% paraformaldehyde, permeabilized with 0.5% Triton X-100 and blocked with PBS supplemented with 1% bovine serum albumin. Samples were stained for AIF and EndoG with the same primary antibodies as for western blotting, followed by Alexa Fluor 488 (A-21206, ThermoFisher Scientific, Carlsbad, CA) labeled anti-rabbit antibodies. Cells were counterstained with DAPI, mounted on slides and photomicrographs recorded using confocal microscopy (Zeiss LSM 700) under 40× oil immersion.

### Subcellular fractionation

Nuclear, mitochondrial and cytosolic fractions were separated using the Mitochondria Isolation Kit for Cultured Cells (89874, ThermoFisher Scientific) according to the manufacturer’s specifications. Briefly, buffers provided with the kit were supplemented with Protease Inhibitor Cocktail (P8340, Sigma) and used to lyse the cells with a Dounce homogenizer, followed by separation by variable speed centrifugation.

### Western blot analysis

Nuclear and mitochondrial protein was extracted with RIPA buffer (89901, ThermoFisher Scientific) supplemented with 1% Protease Inhibitor Cocktail Set I (539131, Merck, Burlington, MA). Samples were incubated on ice and repeatedly vortexed for 10 min, followed by ultrasonication (10 × 30 s at 4 °C) and centrifugation. Protein lysate was electrophoretically separated on SDS-PAGE gels (456–1084, Bio-Rad, Hercules, CA) and transferred to PVDF membranes (88518, ThermoFisher Scientific). Membranes were probed with antibodies for AIF (5318, Cell Signaling, Danvers, MA), EndoG (ab9647, Abcam, Cambridge, MA), NUP98 (2598, Cell Signaling) and β-actin (A2228, Sigma-Aldrich), followed by HRP-anti mouse IgG (474–1806, KPL Inc.) or HRP-anti-rabbit IgG (474–1506, KPL, Gaithersburg, MD) as indicated. Membranes were developed using LumiGLO (7003, Cell Signaling) and a Bio-Rad Chemidoc XRS system; bands were quantified with the Image Lab 5.1 software (Bio-Rad). All measurements were normalized to β-actin and presented as fold change of AIF and EndoG in fractions from smoke exposed cells compared to corresponding clean air controls. Staining for the nuclear marker NUP98 was used to verify purity of the subcellular fractions (cp. Supplementary Fig. 1). Total PARP–1 was extracted from HBE cells and analyzed by western blotting using an anti-PARP–1-antibody (9542, Cell Signaling) as detailed for the cell fractions above.

### PARP–1 Inhibitor

100 μM BMN673 (S7048, Selleckchem, Houston, TX) was added to HBE cultures one hour before exposure to smoke of eight cigarettes. Control cells were given an identical concentration of the diluent DMSO, as found in the experimental samples. Cells were fractionated and processed for western blot analysis three hours after smoke exposure.

### RT-PCR

RNA was extracted from fully matured HBE cells with the AllPrep DNA/RNA Mini kit (80204, QIAGEN AG, Germantown, MD) and treated with DNase I (M0303S, New England BioLabs Inc., Ipswich, MA) before reverse transcription with the iScript cDNA Synthesis Kit (1708891, Bio-Rad). Samples were amplified on a CFX-96 Bio-Rad real time PCR machine using SsoAdvanced universal probes supermix (172–5284, Bio-Rad) and TaqMan primers for PARP-1 and GAPDH as housekeeping gene (Hs00242302_m1 resp. Hs02758991_g1, Thermo Fisher Scientific).

### Statistics

Statistical analysis was performed with Graphpad Prism 5 (GraphPad Software, La Jolla, CA). One sample *t*-tests were used to test whether the data from smoke exposed HBE cells normalized to their clean air control differ from 1, i.e. whether AIF and EndoG content in subcellular fractions from smoke exposed cells differs from their clean air controls. Paired two-tailed *t*-tests were used to test whether BMN673 reduces AIF and EndoG translocation in inhibitor treated cells compared to controls from the same donors. Unpaired two-tailed *t*-tests were used comparing smoke-mediated parthanatos activation in HBE cells from smokers and non-smokers, and the Mann Whitney test to compare PARP–1 expression levels.

## Supplementary information

Supplemental Figure 1

Supplemental Material File #1
