# Supplementary figures and images for: Cigarette smoke activates the parthanatos pathway of cell death in human bronchial epithelial cells
Source: Cell Death Discov. 2019 Aug 5;5:127. doi: 10.1038/s41420-019-0205-3 (PMC6683143; doi:10.1038/s41420-019-0205-3)

**mitochondrial  
fractions**

**nuclear  
fractions**

air

CS

air

CS

NUP98

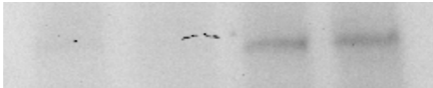

98kDa

b-actin

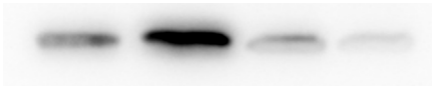

42kDa

Supplement: Supplementary file 1 — Supplemental Figure 1 [file 41420_2019_205_MOESM1_ESM.pdf]
