# Supplementary material for: Cigarette smoke activates the parthanatos pathway of cell death in human bronchial epithelial cells
Source: Cell Death Discov. 2019 Aug 5;5:127. doi: 10.1038/s41420-019-0205-3 (PMC6683143; doi:10.1038/s41420-019-0205-3)
Supplement: Supplementary file 2 — Supplemental Material File #1 [file 41420_2019_205_MOESM2_ESM.docx]

**Fig S1:** HBE cultures were exposed to smoke of 8 cigarettes or an equivalent volume of clean air and fractionated 3 hours after exposure. Proteins were electrophoretically separated, transferred to PVDF membranes and mitochondrial and nuclear fractions were probed for the nuclear marker NUP98 and β-actin.
